# Ecuador’s 2025 presidential election and the disconnection between public health and the political agendas

**DOI:** 10.3389/fpubh.2025.1628203

**Published:** 2025-09-16

**Authors:** Esteban Ortiz-Prado, Enrique Terán, Karla Alejandra Cuenca, Julia Saa Echeverria, Martín Gallardo, María Paz Cadena, Mateo Barriga, Jorge Vasconez-Gonzalez, Juan S. Izquierdo-Condoy

**Affiliations:** ^1^One Health Research Group, Faculty of Health Science, Universidad de Las Americas, Quito, Ecuador; ^2^Colegio de Ciencias de la Salud, Universidad San Francisco de Quito USFQ, Quito, Ecuador

**Keywords:** public health policy, Ecuador, evidence-based policymaking, health equity, political proposals

## Abstract

**Background:**

In Latin America, public health proposals by presidential candidates often lack methodological rigor, limiting their feasibility and impact. Evidence-based planning aligned with national health priorities and disease burden is essential to address critical issues such as chronic diseases, mental health, and healthcare access.

**Objective:**

This study evaluates the methodological robustness of public health policy proposals from 16 Ecuadorian presidential candidates for the 2025–2029 elections. The assessment focuses on key health variables, analyzing the presence of SMART objectives, epidemiological evidence, and alignment with local and global health priorities.

**Methods:**

A systematic evaluation framework was applied to analyze the health components of each candidate’s plan. The study used internationally recognized policy evaluation models, including the CDC’s Six-Step Policy Evaluation Framework and the UK Magenta Book Guidelines. Health variables were weighted based on national priorities, with percentage scores assigned according to alignment with GBD 2021 Ecuador data. Each proposal was assessed for inclusion or omission of these variables, allowing for a comparative ranking of methodological rigor.

**Results:**

The analysis of public health proposals from Ecuadorian presidential candidates revealed significant methodological deficiencies. A total of 81% of proposals lacked SMART objectives, limiting their ability to establish measurable goals. 76% failed to integrate key health determinants such as environmental health, intersectoral collaboration, and research funding. 92% did not include a defined financial strategy, raising concerns about feasibility. Only one candidate (Noboa/Pinto) scored above 50% compliance with the GBD Ecuador 2021 priorities. Mental health and infectious disease prevention were the most frequently addressed topics, while air pollution, food safety, and post-market drug surveillance were largely overlooked. Chronic disease care, environmental sanitation, and vaccine production were among the most underrepresented health priorities.

**Conclusion:**

Public health proposals from Ecuadorian presidential candidates (2025–2029) showed major methodological gaps, with 81% lacking SMART objectives and 92% lacking financial plans. Key areas such as neonatal care and non-communicable disease prevention were often omitted. A more systematic, evidence-based approach is needed, supported by collaboration between policymakers, researchers, and international health agencies.

## 1 Introduction

Public health policies proposed by government candidates play a critical role in shaping national healthcare strategies. However, in many Latin American countries, including Ecuador, these proposals often lack methodological rigor, failing to address key public health determinants effectively. The absence of evidence-based planning, limited integration of epidemiological data, and poor financial structuring weaken the feasibility of these health initiatives (1).

Since 2007, Ecuador began the process of transforming its health sector toward universal and free access to healthcare (2). In 2008, the Constitution of Ecuador recognized the right to health with a systemic approach. That same year, the Constitution also established the State as the guarantor of the right to health through the formulation of policies, plans, and programs (3). Between 2007 and 2016, a total of USD 16.208 billion was invested in health. In 2017, the investment amounted to USD 306 million, which was reduced in subsequent years to USD 201 million in 2018 and USD 110 million in 2019 (3, 4). Later, due to the COVID-19 pandemic and the resulting additional costs, the government invested USD 897 million, of which USD 363 million were allocated to the National Vaccination Plan (5). By 2023, national health spending increased by 2.9%, reaching USD 7.773 billion. According to the 2024 Accountability Report, the most significant actions included investments in medicines, infrastructure, and medical equipment, with a total budget execution of USD 2.742 billion (6, 7).

Currently, the healthcare system faces multiple structural challenges, including persistent shortages of essential medications, which compromise equitable access to treatment (8, 9). These deficiencies are compounded by a lack of medical supplies and substandard working conditions for primary care professionals, all of which negatively impact the quality and continuity of healthcare services (10, 11). Institutional fragility further exacerbates the situation, driven in large part by the frequent turnover of health authorities, which disrupts governance and undermines strategic continuity. This issue is particularly evident within the Ministry of Public Health, where leadership positions have often been assigned to individuals with limited expertise in health policy and management. Notably, between 2023 and 2025, five different health ministers have held office (8, 12, 13). Another pressing issue and priority is the government’s debt to dialysis service providers, which amounts to 250 million USD dólares (14, 15). As a result, these providers lack the necessary medical supplies, which has even led to the death of several patients (16).

In Ecuador, past healthcare reforms have faced challenges in execution due to weak institutional structures, insufficient and irregular resource distribution, leading to unequal access to medical services, especially in rural areas (17). Additionally, the lack of interdisciplinary cooperation and the prioritization of short-term political interests have obstructed long-term advancements in public health. For example, the severe health, social, and economic consequences of the pandemic may persist for decades, due to deficiencies in the poorly functioning healthcare system, worsening social disparities. In the absence of comprehensive and forward-thinking policies, nations may experience extended recovery times, deteriorating public health conditions, and greater financial strain on vulnerable communities (18). To overcome these obstacles, it is crucial to implement evidence-based strategies to improve the efficiency and durability of health policies. It is also important to highlight that good governance enables the delivery of health services in an equitable and sustainable manner. High-quality governance in health policy formulation is a key factor in shaping the improvement of health systems, their functioning, legitimacy, and outcomes (19, 20).

The use of research evidence in public health policymaking is often inconsistent due to barriers such as restricted access to relevant studies, political and ideological influences, and the lack of systematic reviews tailored to local contexts (21, 22). Decision-makers frequently rely on anecdotal evidence or public sentiment rather than robust scientific data, which can result in policies that are misaligned with actual healthcare needs (23). Moreover, the limited application of knowledge translation strategies further impedes the implementation of research-informed policies (24).

Given these methodological deficiencies, there is an urgent need for evidence-based public health proposals. Integrating multidisciplinary evidence, fostering knowledge translation, and utilizing rapid review methodologies can strengthen public health planning (25). Furthermore, aligning policy proposals with national disease burden data, such as the Global Burden of Disease (GBD) study, ensures that healthcare priorities reflect the most pressing population health concerns (26).

**Figure 1:**
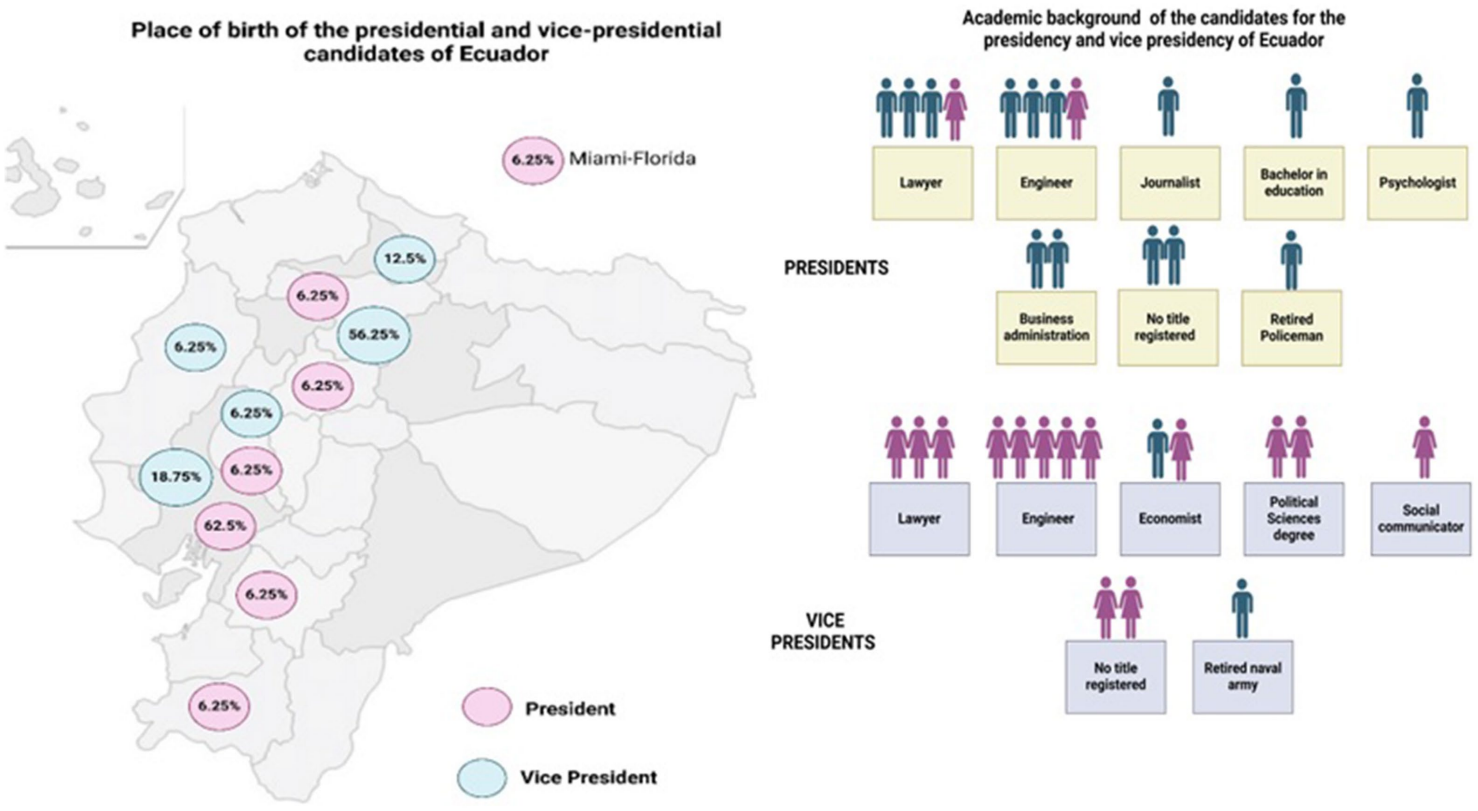
Characteristics of the candidates for the presidency and vice presidency of Ecuador (Created with BioRender.com).

This study assesses the methodological robustness of Ecuadorian presidential candidates (Figure 1) public health policy proposals for the 2025–2029 elections. This study applies internationally recognized policy evaluation frameworks—the CDC’s Six-Step Policy Evaluation Framework and the UK Magenta Book Guidelines—not as rigid protocols, but as methodological guides to assess structure, feasibility, and use of evidence in health policy proposals (27, 28). These frameworks helped inform the analysis of SMART objectives, integration of epidemiological data, financial planning, and clarity of implementation strategies. The findings highlight critical gaps and emphasize the importance of structured, evidence-based policymaking to improve health outcomes in Ecuador.

## 2 Materials and methods

### 2.1 Study design

This study is a qualitative policy analysis assessing the methodological rigor of public health proposals from 16 Ecuadorian presidential candidates for the 2025–2029 election cycle. The study evaluates how well these proposals align with national health priorities and evidence-based policymaking principles. The analysis focuses on the inclusion of structured planning, epidemiological data, financial feasibility, and intersectoral health approaches.

### 2.2 Sample and setting

This study analyzed the official public policy proposals of all 16 presidential binomials registered for the 2025 Ecuadorian national elections. Documents were collected between August and October 2024 from official candidate websites, political party platforms, and publicly accessible campaign materials, including manifestos and downloadable plans. Only proposals that explicitly included public health policy components were eligible for analysis. Materials lacking clear health-related content were excluded.

Ecuador, a country of over 17 million people, presents a complex public health landscape with disparities in access, disease burden, and healthcare infrastructure. Therefore, evaluating presidential candidates’ health policies is crucial to understanding their potential impact on national healthcare outcomes.

### 2.3 Data collection and analysis

Data collection followed a deductive qualitative content analysis framework. Two independent reviewers screened each full-length government plan for health-related content. Explicit policy measures were extracted verbatim and categorized using a structured matrix based on a predefined list of 30 public health policy variables. These variables were selected through a systematic process grounded in Ecuador’s epidemiological profile, as reported in the Global Burden of Disease (GBD) 2021 study. Additional data sources included national hospital admission and mortality records from the Instituto Nacional de Estadística y Censos (INEC, 2023), and regional health priorities outlined by the World Health Organization (WHO) and the Pan American Health Organization (PAHO) (5, 29–31).

The 30 variables were organized into seven thematic domains to ensure comprehensive and non-overlapping coverage of key public health priorities. These domains included health equity, access to healthcare, mental health, infectious disease control, environmental health, chronic disease care, and pharmaceutical and regulatory policy, such as vaccine production and drug surveillance. Only specific and actionable proposals aligned with these predefined variables were included in the analysis. Vague or aspirational statements—for instance, general mentions of “improving health”—were excluded unless they clearly met the established criteria. Each reviewer independently coded the material, and discrepancies were resolved through consensus discussions.

### 2.4 Scoring and weighting system

Each candidate’s proposal was systematically evaluated using a binary scoring system. For each of the 30 predefined public health policy variables, a score of 1 was assigned when the proposal included a clear, specific, and actionable measure; a score of 0 was assigned when the variable was absent or vaguely addressed. This allowed for a standardized and replicable comparison across all proposals. The weighting of each topic was determined based on its relative impact on Ecuador’s health burden.

To account for the relative importance of each variable, weights were assigned based on their estimated contribution to Ecuador’s national health burden, as reported in the GBD 2021 study. Higher-weighted variables included healthcare access (7%), mental health (6%), infectious diseases (6%), chronic disease care (6%), and health equity (5%). Moderate-weighted variables such as environmental health, food safety, epidemic preparedness, and vaccine production were assigned weights ranging from 2 to 5%, reflecting their intermediate impact on public health outcomes.

The final score for each candidate was calculated by summing the weighted scores across all variables, expressed as a percentage of the maximum possible score (100%). This allowed for a comparative ranking of methodological rigor among all 16 proposals.

### 2.5 Statistical analysis

Descriptive statistics were used to summarize the frequency and distribution of public health policy measures across the 16 candidate proposals. Comparative ranking analysis was conducted based on the total weighted scores, enabling the identification of gaps in coverage and methodological inconsistencies. Inter-rater reliability was assessed using Cohen’s kappa statistic, which yielded a value of 0.85, indicating strong agreement between the two independent members of the research team. Any discrepancies in variable classification were resolved through consensus discussions.

### 2.6 Ethical considerations

This study received approval from the local Institutional Review Board (IRB), the Comité de Ética de Investigación en Seres Humanos de la Universidad de Las Américas (CEISH-UDLA), with exemption letter number 2023-EXC-008. CEISH-UDLA determined that the project is exempt from further ethical evaluation, in accordance with current legal regulations. The study relied exclusively on publicly available, non-identifiable government data, without involving human participants.

## 3 Results

### 3.1 General results

The analysis of the public health proposals from the 16 Ecuadorian presidential candidates showed significant differences in their comprehensiveness and alignment with national health priorities. As per the Ecuadorian electoral law, named Democracy code, in its article 3, the state warranties the equal participation of women and men [9a—Consejo Nacional Electoral. Reglamento para la democracia interna de las organizaciones políticas. Quito. 2022 (32)]. Despite of that, the vast majority of the presidential candidates were males (87.5%), and therefore the same number of vice-presidential candidates were females.

Regarding the academic profile of the presidential candidates, there were equal number of lawyers (n = 4) than those with an engineering diploma. However, there were still two candidates with no title, and one retired policeman. Similarly, for the vice-presidency, there were more candidates with an engineering diploma (n = 5), although it was in marketing, business management, or information technology; followed by lawyers (n = 4). There were also two candidates with no registered title and one retired army candidate (Figure 1).

The total scores for each candidate ranged from 53 to 98%, based on the number of addressed variables weighted according to their health burden. Escala/Terán achieved the highest score at 98%, covering nearly all evaluated variables. Tillería/Rosero had the lowest score at 53%, addressing the fewest health-related concerns.

Access to healthcare was present in 14 proposals, while mental health and infectious disease control were included 13 and 9 proposals, respectively. Tobacco control was presented only in 1 proposal, and post-market drug surveillance and counterfeit medicine control were included in the 2 and 5 proposals, respectively. Vaccine production appeared in only in 3 proposals, and chronic disease care was inconsistently addressed.

In Table 1 are summarized the concrete public health proposal declared in the official governmental plans of the binomial candidates.

**Table 1:** Public health proposal declared in the official governmental plans of the binomial candidates.

Thematic area	Cucalón / Larrea	Cueva / Reyes	Tillería / Rosero	Noboa / Pinto	Gonzales / Borja	Gonzalez / Moncayo
Access to health care	Provision of resources, improvement of infrastructure, and reduction of waiting times	Reduce the gap in access to health services, especially for the most vulnerable populations.	A model based on universality, the creation of a health network that integrates public and private clinics, the renovation of hospitals, and the construction of 10 new ones	Increase in the hiring of healthcare personnel.	Promotion of accessible, resilient, and equitable health systems and reform of social security.	It does not mention
Mental health	Creation of specialized centers and suicide prevention programs	Creation of specialized centers for the prevention and treatment of mental disorders, along with suicide prevention programs.	It does not mention	Emotional and psychological support and assistance programs	It does not mention	Creation of wellness programs
Child malnutrition	National strategy to reduce and prevent chronic malnutrition	It does not mention	It does not mention	Promote healthy and nutritious foods by boosting local production, with a focus on food security. Implement nutritional counseling programs and promote breastfeeding	Sustainable food systems that ensure food security, diversity of nutritious foods, and fair access to them.	Promote food and nutritional security by ensuring adequate nutrition for children, including a school breakfast program.
Sanitation,wáter/ Environment	New infrastructure for sanitation, waste management, and promotion of recycling	Modernize sanitation infrastructure and drinking water distribution systems.	Improve air and water quality and reduce pollution. Expand access to drinking water by increasing infrastructure and sanitation systems.	Reduction of greenhouse gas emissions; sustainable management of water resources through the protection of aquatic ecosystems; and research in renewable and clean technologies.	Emergency plan to recover our natural heritage, thereby addressing pollution and improving environmental health.	Creation of a national network for monitoring air and water quality in urban and rural areas.
Sexual and reproductive health	It does not mention	Greater investment in sexual and reproductive health and improvement of the school curriculum to include education on teenage pregnancy.	It does not mention	Increase the percentage of people living with HIV who know their serological status and are receiving treatment. Promote access to sexual and reproductive health through the provision of family planning programs, access to information and contraceptive methods, and reproductive education.	It does not mention	Promote food and nutritional security by ensuring adequate nutrition for pregnant women.
Vaccination	It does not mention	It does not mention	It does not mention	Mass vaccination campaigns	It does not mention	It does not mention
Chronic diseases	Follow-up and specialized care plan	It does not mention	It does not mention	Improvement and modernization of clinics and health centers, with a better intersectoral approach to health and the capacity to treat chronic and catastrophic diseases.	The infrastructure of health units will be strengthened	It does not mention
Medications	Establishment of a transparent system for the purchase of medicines	Make the medicine procurement process transparent, reduce intermediaries, and ensure supply throughout the country.	It does not mention	It does not mention	Promote the production of generic medicines through the national industry within the framework of regional integration.	Promote national drug production through agreements with private pharmaceutical companies. Reform medicine procurement procedures by eliminating intermediaries and making direct purchases from manufacturers.
Digitalization	Digitalization of medical records	It does not mention	Telemedicine	It does not mention	It does not mention	Implement digital transparency platforms where contracts, acquisitions, and health system budgets are publicly recorded.
Medical training	It does not mention	Granting of at least 4,000 scholarships.	It does not mention	Development of continuing education programs.	Revitalize the country’s public and flagship universities, implement scholarship programs abroad, and promote excellence and quality.	It does not mention
Violence / Security	It emphasizes security and violence prevention, aiming to reduce the burden of homicides on the health system	It describes violence and insecurity as its primary concern.	Increase the security budget by 20% to combat crime.	It does not mention	It does not mention	It does not mention
Omitted regional health problems	Neonatal disorders, road traffic injuries, lower respiratory infections, falls, low back pain, HIV/AIDS, congenital defects, headache disorders, and age-related hearing loss, vaccination, epidemiological surveillance, tobacco prevention, safe abortion, food safety, drug control, and teenage pregnancy	Neonatal disorders, ischemic heart diseases, road traffic injuries, lower respiratory infections, strokes, diabetes, falls, chronic kidney diseases, cirrhosis, low back pain, headache disorders, COVID, congenital defects, vaccination programs, chronic disease care, epidemiological surveillance, drug addictions, safe abortions, food safety, chronic malnutrition, electronic medical records, and health research.	Sex education, teenage pregnancies, safe abortions, prevention and treatment of drug addictions, access to medicines, epidemiological surveillance, vaccination, medical training, chronic disease care, child malnutrition, HIV/AIDS, and food safety; neonatal disorders, ischemic heart diseases, road traffic injuries, strokes, falls, diabetes, lower respiratory infections, chronic kidney diseases, age-related hearing loss, COVID, congenital defects, self-harm, and headache disorders.	Generic drug production, control of drug smuggling and post-marketing surveillance are not addressed; nor is smoking prevention mentioned—only alcoholism and addictions through rehabilitation centers.	Infectious diseases, mental health, HIV/AIDS, reproductive health care, generic drug production, use of electronic systems, vaccine production, post-marketing surveillance of medicines, prevention of smoking and other addictions such as alcoholism, burden of disease due to homicides, and safe abortion are not mentioned	Teenage pregnancy, safe abortion, the burden of disease due to homicides, treatment of addictions such as alcoholism, smoking, and other illicit substances are not addressed. The plan also fails to mention a post-marketing surveillance system for medicines or how the health plan will be financed. Furthermore, it does not include strategies for the care of chronic diseases, HIV/AIDS, obesity, or infectious diseases. The plan does not specify how they will address the issue of health inequity
Deficiencies	Once the medications have been acquired, no specific measures are outlined to control smuggling and subsequent commercialization.	The area of sexual and reproductive health lacks information on HIV/AIDS.	They do not specify how long the mental health programs will last, nor how frequently the campaigns will be carried out.	The techniques for reducing greenhouse gas emissions are not clearly explained, nor are the associated implementation strategies.	It does not establish measures for price control and the sale of medicines following their national production.	It makes no reference to training programs on the use of the digital platforms it proposes to create.
References	(42)	(43)	(44)	(45)	(46)	(47)

### 3.2 Alignment with local and global health priorities

**Figure 2:**
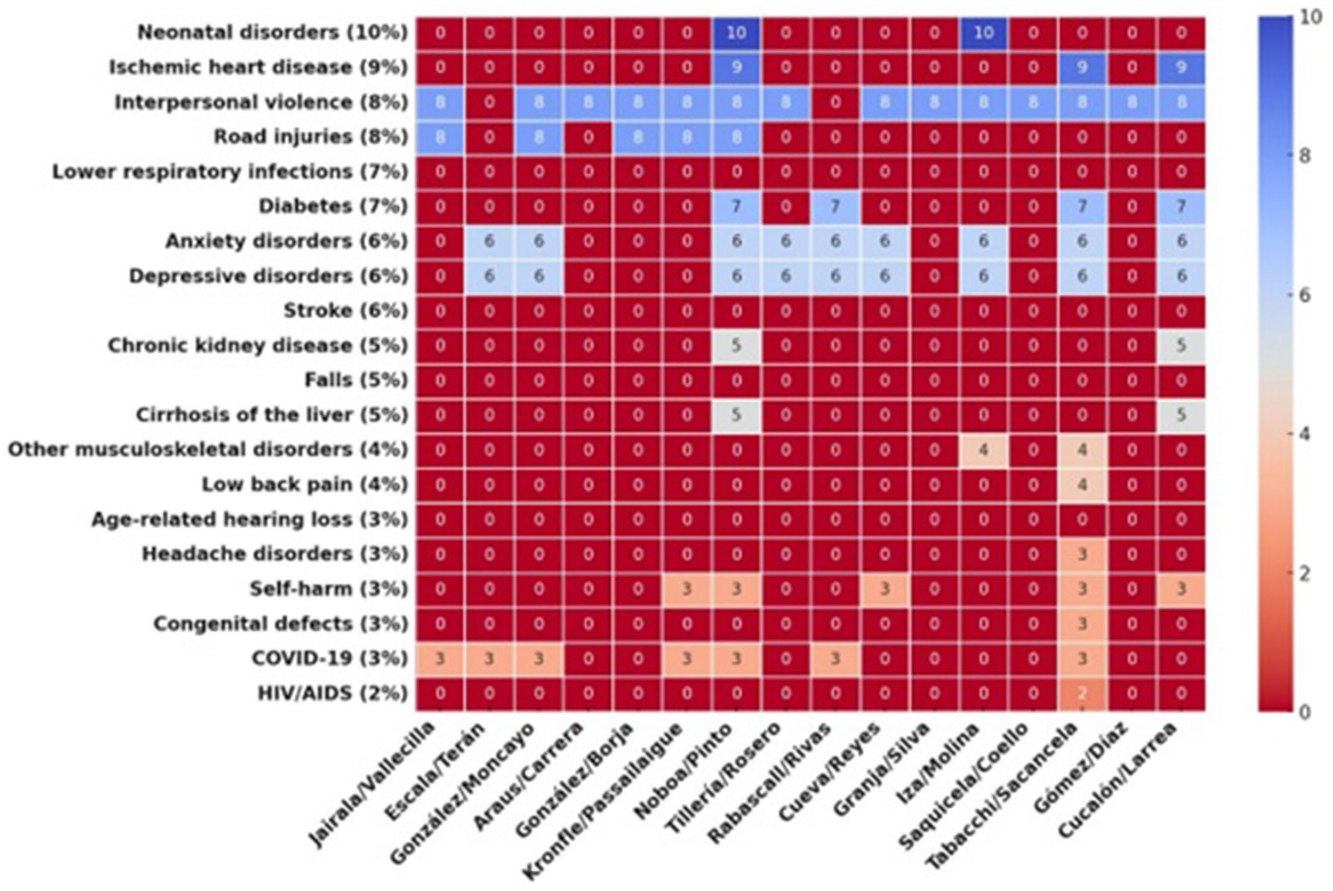
Heatmap displaying the burden of disease distribution across different candidates. The color scale transitions from red (indicating lower values or absence) to deep blue (representing higher values and greater burden). Candidate names are bold and right (Created with Prisma chart).

Among the leading causes of morbidity and mortality in Ecuador, neonatal disorders, ischemic heart disease, and interpersonal violence were the least addressed. Only Noboa/Pinto and Inza/Molina included neonatal care interventions, while preventive strategies for cardiovascular diseases were largely absent (Figure 2). Interpersonal violence was covered in 14 proposals, and road injuries were included in five. Lower respiratory infections and stroke were not mentioned in any proposals.

Non-communicable diseases such as diabetes, anxiety disorders, and depressive disorders were included 4, 9 and 9 proposals, respectively. Mental health was a frequently addressed topic included in 13 proposals, and COVID-19 appeared in 7 proposals. HIV/AIDS prevention and treatment were included in only one proposal.

### 3.3 Addressing local health needs

**Figure 3:**
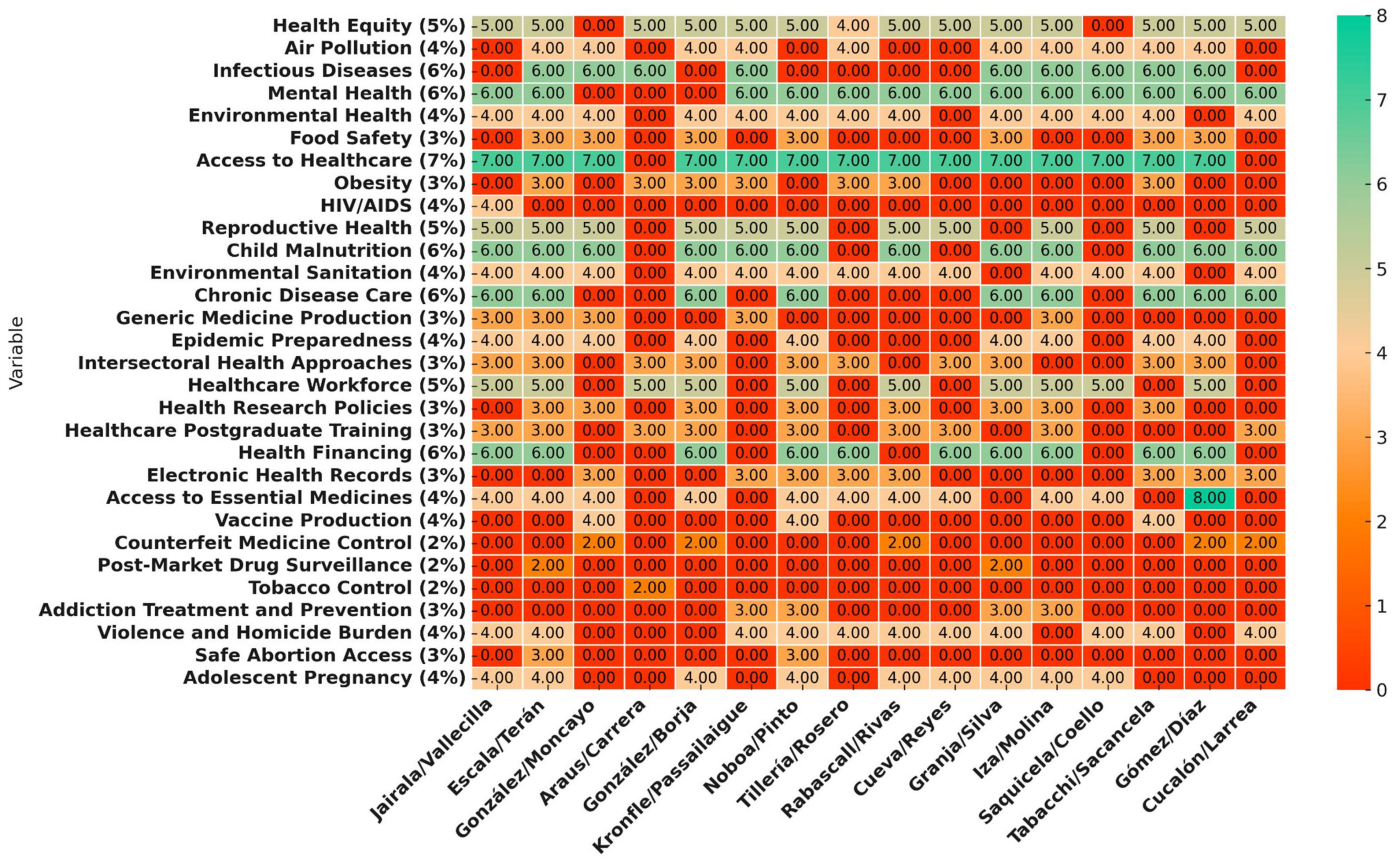
Heatmap displaying the distribution of variables across different candidates. The color scale transitions from red-orange for lower values to turquoise-green for higher values, with dark orange representing zero values. Candidate names are bold and right (Created with Prisma chart).

Healthcare workforce development was addressed in most proposals, while funding for health research and postgraduate training was included in fewer than half. Environmental and food safety policies were inconsistently covered, and air pollution was included in fewer than half of the proposals. Pharmaceutical policy planning varied, with fewer than half of the proposals including access to essential medicines. Vaccine production was mentioned in only a few proposals (Figure 3). Healthcare financing was covered in most proposals, but few included clear strategies for budget allocation.

### 3.4 Overall assessment

**Figure 4:**
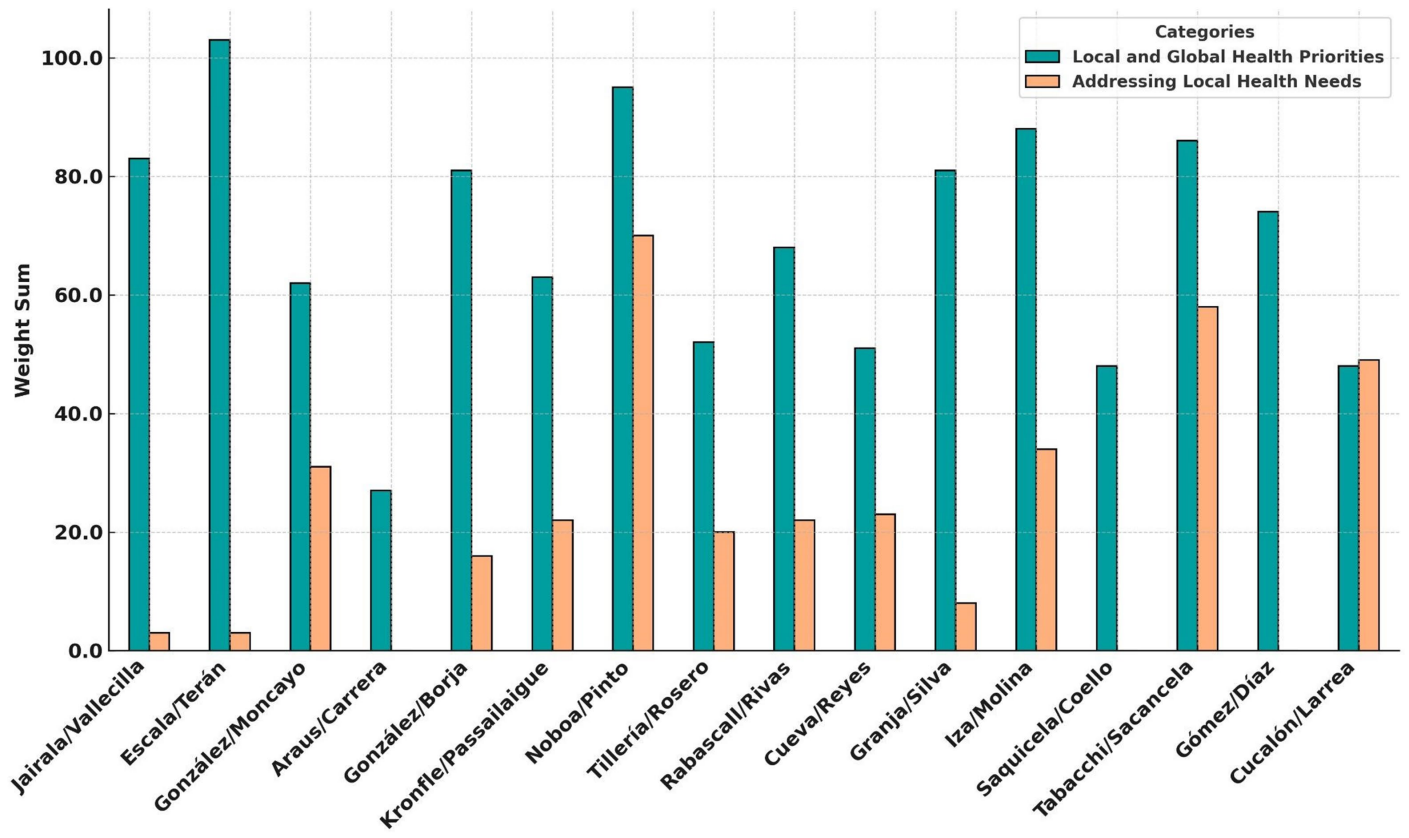
Comparison of local and global health priorities vs. addressing local health needs among presidential candidates. The turquoise bars represent the weight sum for prioritization of local and global health issues, while the peach bars represent the weight (Created with Prisma chart).

Candidates with higher methodological scores used structured frameworks, epidemiological data, and evidence-based interventions. Those with lower scores relied on general statements without clear implementation plans or measurable objectives. Coverage discrepancies indicated a lack of a standardized, evidence-based approach to public health policymaking (Figure 4).

Each of the methodological criteria defined in the study—structured planning, epidemiological data, financial feasibility, and intersectoral approaches—was used as part of the evaluation matrix. These dimensions were assessed comparatively across proposals, and their influence is reflected in the scoring system and visualized in Figure 4.

The findings showed that many proposals lacked alignment with Ecuador’s health burdens. Key areas such as tobacco control, vaccine production, and chronic disease management were frequently omitted, highlighting the need for more comprehensive and data-driven public health policies.

## 4 Discussion

The analysis of public health proposals from candidates in Ecuador’s 2025 presidential election reveals substantial methodological deficiencies that compromise both the feasibility and effectiveness of the proposed policies. A central issue is the limited use of research-based evidence in the decision-making process. Many proposals lack references to epidemiological data, systematic reviews, or burden-of-disease assessments, thereby weakening their scientific foundation. This reflects a broader trend in global public health policymaking, where decision-makers often face barriers in accessing and applying research evidence within policy frameworks. The persistent disconnect between academic researchers and political actors—as well as the perception of scientific evidence as bureaucratic or impractical—contributes to the absence of structured, data-driven strategies in many political health proposals.

Our analysis shows that a multidisciplinary approach to the development of these proposals was largely absent in most candidate plans. Effective evidence-based decision-making requires the integration of multiple scientific disciplines, including epidemiology, environmental health, and behavioral sciences (33, 34). However, many proposals failed to incorporate cross-sectoral perspectives, instead emphasizing isolated policy measures that overlook broader social determinants of health. The absence of systematic reviews and meta-analyses adapted to Ecuador’s health priorities further exacerbates these methodological shortcomings. This pattern reflects challenges common to other low- and middle-income countries, where local health research infrastructures are often underfunded and underutilized.

The lack of robust evidence-based policymaking underscores the urgent need for structured frameworks that ensure policy proposals are grounded in empirical data. A major limitation is the frequent reliance on broad, generic statements that lack clear implementation pathways or measurable objectives. This is particularly evident in the absence of SMART (Specific, Measurable, Achievable, Relevant, and Time-bound) criteria in most proposals, making it difficult to assess policy effectiveness over time. Moreover, the widespread lack of financial planning further calls into question the feasibility of these proposals, as policies without defined budgets are unlikely to be implemented effectively.

Improving public health policymaking in Ecuador will require better integration of governance principles, scientific evidence, and strategic communication (23, 35). Effective health proposals must incorporate not only epidemiological data but also address key governance challenges, such as intersectoral coordination and regulatory enforcement (36). Internationally, knowledge translation strategies have been developed to bridge the gap between research and policy. In the Ecuadorian context, this could include institutional mechanisms that facilitate collaboration between public health researchers and policymakers, ensuring that proposals are informed by high-quality data and systematic evaluations.

Our findings also indicate that most candidate proposals fail to align with Ecuador’s current disease burden or global health priorities. Although mental health and infectious diseases were frequently mentioned, critical areas such as neonatal disorders, ischemic heart disease, and stroke prevention were largely neglected. This misalignment suggests that policy planning is not sufficiently informed by the country’s epidemiological profile. Particularly concerning is the limited focus on chronic disease management, despite the growing impact of non-communicable diseases in Ecuador.

In addition, vaccine production, pharmaceutical regulation, and health system resilience received minimal attention, despite their importance in ensuring national health security. Local vaccine production offers multiple benefits, including a stable supply, reduced dependence on imports, and increased readiness in the face of global shortages. However, Ecuador has experienced a notable decline in vaccination coverage since the discontinuation of domestic vaccine manufacturing, especially for BCG and DTP vaccines (37). The omission of tobacco and e-cigarette control policies, as well as post-market drug surveillance, further reflects a broader neglect of long-term regulatory and preventive health measures (38). This pattern suggests a tendency to prioritize short-term political gains over evidence-based, sustainable health strategies.

To enhance the methodological rigor of public health proposals, it is essential to strengthen the integration of scientific evidence into policy development. Future proposals should incorporate systematic reviews, use current epidemiological data, and include detailed financial planning to ensure feasibility. The development of a standardized framework for evaluating health policy proposals could also improve consistency and alignment with national priorities (39–41). Tools such as rapid review methodologies and Delphi consensus techniques—already applied successfully in other countries—may serve as useful models for improving the design and evaluation of evidence-based public health policies in Ecuador.

The findings highlight the need for a shift in the political approach to health policymaking, moving from broad, aspirational goals to structured, evidence-based strategies that are actionable and measurable. Addressing these gaps will require increased collaboration between policymakers, public health researchers, and regulatory institutions to ensure that future proposals align with Ecuador’s pressing health needs while adhering to global best practices.

## 5 Limitations

This study has several limitations that should be considered when interpreting its findings. First, the analysis relied exclusively on publicly available government plans, which may not fully reflect the entire scope of each candidate’s health policy agenda. Some candidates may have had additional, unpublished proposals or internal strategies not captured in this review. Moreover, the absence of direct communication with candidates or their policy teams limits our understanding of the rationale behind specific policy choices, contextual constraints, or implementation considerations. Importantly, the policy proposals analyzed reflect pre-electoral declarations that are subject to change once candidates are elected and must develop official government plans. Therefore, the dynamic nature of political program refinement was not captured in this cross-sectional study.

Second, the level of detail and clarity varied significantly across the proposals. While some candidates provided structured frameworks with measurable objectives, others included only broad or aspirational statements. This variation may have influenced the scoring process, as more detailed proposals were more likely to explicitly address the predefined variables. Although a binary scoring system was applied uniformly, the inherent asymmetry in proposal quality introduces potential bias.

Third, the assignment of weights to the 30 public health variables was based on expert judgment informed by epidemiological data from the GBD 2021 study and national statistics. However, the process did not involve a formal consensus method such as a Delphi panel and therefore introduces a degree of subjectivity. While grounded in evidence and public health expertise, the prioritization scheme may not fully reflect the political, economic, or institutional constraints affecting policy feasibility in Ecuador.

Fourth, although the study emphasizes the use of scientific evidence in policy formulation, we recognize that other factors—such as governance capacity, political will, stakeholder influence, and budget availability—may exert greater relative weight in determining policy effectiveness. The mere presence of evidence-informed content does not ensure implementation success. Future research should explore these political and institutional determinants in greater depth.

Finally, the study employed a cross-sectional design that captures the content of the proposals at a single point in time. As such, it does not assess how these policies may evolve, be revised, or ultimately be implemented if the candidates are elected. The absence of a longitudinal component limits the ability to examine real-world impact or policy sustainability.

Despite these limitations, this study provides a structured evaluation of the methodological rigor of health proposals and highlights critical gaps in evidence-based policymaking. Addressing these challenges will require greater transparency in policy formulation, stronger integration of scientific evidence, and improved mechanisms to assess the long-term impact of proposed health interventions.

## 6 Conclusion

This evaluation of public health proposals from Ecuadorian presidential candidates reveals substantial methodological shortcomings. Many plans lacked structured, evidence-based approaches, with limited use of standardized frameworks, few SMART objectives, and insufficient financial planning—factors that collectively undermine the feasibility and effectiveness of the proposed interventions. Although frequently discussed issues such as healthcare access and mental health were commonly addressed, several critical areas—such as neonatal care, ischemic heart disease, and stroke prevention—were largely neglected. These omissions point to a lack of alignment with both national health needs and global disease burden priorities.

Candidates who achieved higher methodological scores were those whose proposals incorporated epidemiological data, structured planning, and clear financial strategies. In contrast, candidates with lower scores often relied on general, non-specific statements that lacked measurable goals or defined implementation pathways. The overall inconsistency across proposals underscores the urgent need for a more systematic, evidence-informed approach to public health policymaking in Ecuador.

To enhance the quality and impact of future proposals, it is essential to strengthen the integration of scientific evidence into political health agendas. Policymaking should be aligned with Ecuador’s current epidemiological profile and give priority to underrepresented areas such as chronic disease management, environmental health, and vaccine production. Moreover, the tendency to favor politically expedient or short-term solutions over long-term, evidence-based strategies must be addressed to ensure sustainable improvements in population health.

Improving public health policy in Ecuador will require stronger collaboration among policymakers, public health researchers, and international organizations. This includes the development of institutional mechanisms to evaluate policy proposals based on official data sources—such as those from the INEC or the Ministry of Public Health—and to ensure that political plans are grounded in scientific rigor and national health priorities.

## Data Availability

The original contributions presented in the study are included in the article/supplementary material, further inquiries can be directed to the corresponding author.
